# KRAS-specific antibody binds to KRAS protein inside colorectal adenocarcinoma cells and inhibits its localization to the plasma membrane

**DOI:** 10.3389/fonc.2023.1036871

**Published:** 2023-03-27

**Authors:** Kuen Kuen Lam, Yee Syuen Low, Michelle Lo, Michelle Wong, Choong Leong Tang, Emile Tan, Aik Yong Chok, Isaac Seow-En, Siew Heng Wong, Peh Yean Cheah

**Affiliations:** ^1^ Department of Colorectal Surgery, Singapore General Hospital, Singapore, Singapore; ^2^ JW Bioscience Pte. Ltd., Singapore, Singapore; ^3^ Saw Swee Hock School of Public Health, National University of Singapore, Singapore, Singapore; ^4^ Duke-NUS Medical School, National University of Singapore, Singapore, Singapore

**Keywords:** KRAS, antibody therapeutics, metastatic colorectal cancer, SOX9, *ex vivo* culture

## Abstract

Colorectal cancer (CRC) is the third highest incidence cancer and a leading cause of cancer mortality worldwide. To date, chemotherapeutic treatment of advanced CRC that has metastasized has a dismayed success rate of less than 30%. Further, most (80%) sporadic CRCs are microsatellite-stable and are refractory to immune checkpoint blockade therapy. KRAS is a gatekeeper gene in colorectal tumorigenesis. Nevertheless, KRAS is ‘undruggable’ due to its structure. Thus, focus has been diverted to develop small molecule inhibitors for its downstream effector such as ERK/MAPK. Despite intense research efforts for the past few decades, no small molecule inhibitor has been in clinical use for CRC. Antibody targeting KRAS itself is an attractive alternative. We developed a transient *ex vivo* patient-derived matched mucosa-tumor primary culture to assess whether anti-KRAS antibody can be internalized to bind and inactivate KRAS. We showed that anti-KRAS antibody can enter live mucosa-tumor cells and specifically aggregate KRAS in the cytoplasm, thus hindering its translocation to the inner plasma membrane. The mis-localization of KRAS reduces KRAS dwelling time at the site where it tethers to activate downstream effectors. We previously showed that expression of SOX9 was KRAS-mutation-dependent and possibly a better effector than ERK in CRC. Herein, we showed that anti-KRAS antibody treated tumor cells have less intense SOX9 cytoplasmic and nuclear staining compared to untreated cells. Our results demonstrated that internalized anti-KRAS antibody inhibits KRAS function in tumor. With an efficient intracellular antibody delivery system, this can be further developed as combinatorial therapeutics for CRC and other KRAS-driven cancers.

## 1 Introduction

Colorectal cancer (CRC) is one of the leading cancers in the developed world with almost 900,000 deaths annually (1, 2). The 5-year age-standardized observed survival is only 60% and 10% for lymph-node-involved Stage III and distal organ-involved Stage IV CRC respectively (3). Currently, the success rate of first line chemotherapy of 5-Fluorouracil and oxaliplatin for metastatic CRC is less than 30% (4, 5). Patients who do not respond to this first line therapy and have wild-type *KRAS* gene are sometimes given the anti-EGFR (epidermal growth factor receptor) therapy, Cetuximab or Panitumumab. However, the overall survival is only 4.6% to 12.3% for Cetuximab monotherapy and 6.9% to 18.9% for Cetuximab in combination with chemotherapy for colorectal cancer, and almost all therapy recipients eventually develop resistance to this second line therapy due to the selective pressure for activating mutation of proto-oncogene *KRAS*, downstream of the EGFR signaling pathway (6–8). Although immune checkpoint blockade therapy has some success with microsatellite-unstable CRCs, most (80%) sporadic CRCs are microsatellite-stable and refractory to such therapy (9). There is thus an urgent need to develop better therapeutics for this deadly cancer. Oncogenic RAS has been shown to be essential for tumor maintenance and KRAS mutation in CRC is associated with metastasis and poor prognosis (10–13).

Despite KRAS displaying a central role in CRC tumorigenesis and possibly metastasis, direct inhibition of KRAS is exceptionally challenging as it is not receptive to inhibitor docking (14–16). Approximately 50% of CRC harbors KRAS oncogenic mutations and our unpublished findings show that *KRAS* mutations is associated with metastasis (17). To date, the only approved direct KRAS inhibitor is Sotorasib (AMG 510) which specifically targets KRAS p.Gly12Cys in non-small-cell lung carcinoma by forming a covalent bond with the 12-cysteine (18). Nevertheless, KRAS p.Gly12Cys mutations are rare in CRC. Alternative strategies like inhibition of farnesyl transferase which prevents KRAS C-terminal prenylation, required for inner plasma membrane localization where KRAS functions, were unsuccessful as farnesyl transferase are functionally replaced by geranylgeranyl transferase (19, 20). Efforts were then diverted to inhibit KRAS downstream targets Raf/MEK/ERK, also known as mitogen-activated protein kinase (MAPK) and PI3K/Akt (21, 22). Despite extensive efforts in research and clinical trials over the past three decades, none of these inhibitors have progressed to clinical use for CRC. In fact, to date, only one clinical trial (NCT02788279) has progressed past Phase 2 [reviewed by Xie, Chen (23)]. Our recent study also reported the absence of MAPK and PI3K/Akt pathways activation in CRC tumors compared to matched normal mucosa indicating that phosphorylated ERK/AKT may not be the appropriate downstream effectors to repress *KRAS* signaling (24).

Antibody targeted therapy has much reduced toxicity than small-molecule therapies (25). However, as KRAS is an intracellular protein, it is a challenge to transport the antibody into the cells. Nonetheless, there are several reports of autoantibodies that can naturally penetrate into cells as reviewed by Ruiz–Arguelles and Alarcon–Segovia (26). These autoantibodies can be found in the serum of autoimmune patients which likely contribute to disease progression, for instance Anti-U1snRNP IgG penetrate into subsets of human T lymphocytes, anti-dsDNA (double stranded DNA) IgG penetrate into human lymphocytes and kidney glomerular cells, both trigger active cell death and tissue damage (27–31). Anti-dsDNA 3E10 IgG from systemic lupus erythematosus-prone mice can also be internalized into the cytosol, and its derivatives like TMab4 have shown cell-penetrating properties (32–34).

We needed a model for the preliminary study of anti-KRAS antibody internalization. While CRC cell lines are the most common experimental model, most cell lines are highly culture adapted thus often do not reflect biological characteristics of CRC in patients. Ronen, Hayat (35) reported that many commonly used CRC cell lines (e.g., HT-29 and LOVO) failed to cluster with human CRC tissue based on multi-omics studies (copy number alterations, transcriptome, somatic mutations, gene methylation), indicating their divergence from CRC in biological characteristics. *In vivo* studies of animal models, mostly mouse models of CRC may not mimic the human disease process and requires a long time, and hence not ideal for proof-of-concept studies (36). While patient-derived xenograft models consider patient-to-patient tumor variation, the environment for the tumor growth is from the mice rather than human, and further differences in environment is contributed by heterotopic models, and similarly requires a long lead time to propagate. Patient-derived organoids are decent models for CRC but is technically challenging and time consuming, thus not suitable for preliminary screening tests, like our current proof-of-concept study. Organoids have other disadvantages like accessibility of cells to treatment due to their three-dimensional structure, the need to start from stem cells and failure of the stem cells to terminally differentiate in the organoid (37).

In this study, we developed an *ex vivo* cell culture of crypt epithelial cells derived from patients’ CRC tumor and matched mucosa tissues. In this *ex vivo* culture system, the culture is transient, viable for 3 days. This minimizes culture adaptation such that cells largely retain their original biological characteristics, thus a more accurate representation of human CRC. Both the tumor and matched mucosa crypt epithelial live cells were treated with anti-KRAS antibodies. The 2-dimensional nature of this culture system allows us to clearly visualize the internalization of anti-KRAS antibodies by confocal microscopy. The data can be correlated to the patient’s clinicopathological features (such as age, gender, tumor stage, site, and differentiation). The availability of archived human matched mucosa-tumor tissues of the same patients would enable us to profile the genomics, transcriptomics and metabolomics, whenever necessary to elucidate the pathways involved and better understand the biology. The histology of the tumor can also be studied to correlate KRAS internalization and effect on cell viability and KRAS downstream pathway activation. Hitherto, tumor heterogeneity has hampered therapeutics considerably. The availability of samples that are simultaneously cultured and archived would enable more systematic experimentation that could potentially throw more light on inter-tumor heterogeneity and genetic variability of disease subgroups that would no doubt contribute eventually to therapy.

Herein, we showed that anti-KRAS antibodies can be internalized in the *ex vivo* cultured matched mucosa-tumor cells. We showed that most of the internalized anti-KRAS antibodies are localized in the cytoplasm and not endosomes, and that the antibody altered endogenous KRAS localization from the inner plasma membrane to the cytoplasm in tumor cells harboring KRAS p.Gly12Val mutation. As expected, the treatment of mucosa and tumor cells with anti-KRAS antibodies led to a decrease in SOX9 expression.

## 2 Materials and methods

### 2.1 Colon crypts isolation and culture

All colorectal cancer tissue samples were collected from freshly resected colon or rectum. A piece of 1 x 1 cm tumor and a strip of 5-10 x 1 cm of mucosa samples were cut using a scalpel blade and a pair of dissection scissors, respectively. The mucosa sampled is as far from the tumor as allowed by internal guidelines, 2 cm from the resection margin (**Supplementary Figure 1A**). The tissues were transported in phosphate-buffered saline (PBS; 10 mM sodium phosphates, 2.68 mM KCl, 140 mM NaCl) back to the laboratory. Any tumor sample with diameter 2 cm or greater, collected within an hour of surgery from consented patients is included in the study. Tumor tissue which is 2 cm or less in diameter and/or is necrotic is excluded. The study methodologies follow guidelines set by the Human Biomedical Research Act, Ministry of Health, Singapore. The study was also approved by the SingHealth Centralised Institutional Review Board (CIRB project number 2018/2837) and conform to the standards set by the Declaration of Helsinki.

The extraction of crypts from the colorectal tissues for cell culture was adapted from the protocol by Booth, Dove (38). The tumor and mucosa tissues were washed with PBS for 3 times and sterilized in bleach (Clorox, 5.25% sodium hypochlorite) diluted to 0.09% (~0.005% sodium hypochlorite) in PBS at room temperature for 20 min. The tumor and matched mucosa tissues were washed with PBS 3 times. To dislodge crypts of the tissues, the tumor and matched mucosa tissues were first incubated in chelating buffer composed of 0.5 mM EDTA and 0.05 mM DTT in PBS, for 15 minutes in 37°C, then washed with PBS. The debris and top crypts were dislodged by shaking the tissues in PBS vigorously for 15 s, 2 times, and were then discarded. This chelation-dislodging process was repeated for 2 more times in increasing strengths of chelating buffer of 1 mM EDTA with 0.05 mM DTT, then finally 3 mM EDTA with 0.5 mM DTT in PBS for mucosa; and only one more time using the latter buffer for tumor as tumor crypts are easier to dislodge (**Supplementary Figure 1B**). The dislodged crypts were passed through a 40 µm cell strainer (SPL Life Sciences, Gyeonggi-do, Korea) which traps the crypts while allowing single cells to flow through. The crypts were then recovered from the cell strainer in cell culture medium consisting of DMEM (Dulbecco`s Modified Eagle Media; Lonza, Basel, Switzerland) supplemented with 10% FBS (fetal bovine serum; Hyclone, UT, USA) and 2X Pen-Strep (Penicillin-Streptomycin; Thermo Fisher Scientific, MA, USA), then pelleted down by centrifugation at 200x g, 5 min. The pelleted tumor and mucosa crypts were resuspended in cell culture medium and seeded onto coverslips coated with Matrigel (Corning, NY, USA) diluted 1:10 in DMEM with 2X Pen-Strep for 1 hour at 37°C, 5% CO_2_ prior to seeding.

### 2.2 Fixation, permeabilization and immunofluorescent staining

The cells attached to coverslips were fixed with pre-chilled methanol for 5 minutes at -20°C. Cells were washed with cold PBS with calcium and magnesium (PBSCM; PBS with 0.5 mM MgCl_2_ and 0.5 mM CaCl_2_) 5 times for 5 minutes each wash, then permeabilized with PBSCM with 0.1% Triton-X-100 (PBSCM-T) for 30 min at room temperature. The cells were then blocked-in blocking buffer (5% Goat Serum, 5% FBS, 3% BSA in PBSCM) for 1 h at room temperature. Thereafter, they were incubated with primary rabbit polyclonal anti-KRAS antibody (Thermo Fisher Scientific, cat# PA5-27234) at 20 µg/mL, mouse anti-EEA1 antibody (ab70521, Abcam) at 1:200 dilution or anti-SOX9 antibody (Sigma-Aldrich cat# AMAB90795) at 1:200 in blocking buffer at 4°C overnight. After incubation, cells were washed with PBSCM-T for 3 times with 5 minutes interval each. The cells were then incubated in secondary antibodies: FITC conjugated goat anti-rabbit IgG, (Thermo Fisher Scientific cat# 31583) at 1:50, Alexa Fluor 488 conjugated goat anti-rabbit IgG (Cell Signaling Technology, MA, USA, cat# 4412) at 1:500 or Alexa Fluor 555 conjugated goat anti-mouse IgG at 1:500 (Cell Signaling Technology cat# 4409) diluted in blocking buffer. Coverslips were mounted with ProLong™ Gold Antifade mounting medium with DAPI (Thermo Fisher Scientific) or VECTASHIELD^®^ PLUS antifade mounting medium with DAPI (Vector Laboratories, CA, USA) onto microscope glass slides.

### 2.3 Treatment of *ex vivo* culture cells with anti-KRAS antibodies

Rabbit polyclonal anti-KRAS (Thermo Fisher Scientific cat# PA5-27234), rabbit monoclonal anti-Ras (Cell Signaling Technology cat# E4K9L) or mouse monoclonal anti-KRAS (Thermo Fisher Scientific cat# 415700) antibodies were diluted to 20 µg/ml in antibody incubation buffer (DMEM + 1% FBS + 25 mM HEPES + 2X Pen-Strep), or vice versa. Rabbit anti-KRAS polyclonal IgG antibody (dialyzed) and rabbit monoclonal anti-Ras IgG antibody (in PBS without preservatives). The cells attached onto coverslips were washed with 2 ml of cell culture medium for 3 times, with 200 rpm, 5 min of shaking on an orbital shaker each time to thoroughly remove cell debris and sticky dead cells. 80 µl of diluted antibody was added to the center of each well of a new 6-well culture plate, then the coverslip with cells facing downward is placed atop of the diluted antibody, allowing the latter to spread, and come in contact with the cells. The samples were incubated at 33°C, 10% CO_2_ for 16 h. For co-localization experiments, coverslips were processed as in sn2.2< h>ectio above. The epitopes of the anti-KRAS and anti-Ras antibodies are listed in **Supplementary Table 1**.

### 2.4 Confocal microscopy imaging and image processing

Brightfield and phase contrast images were taken with Nikon (Tokyo, Japan) Eclipse Ti inverted microscope. Confocal fluorescence microscope images were acquired with the Nikon A1 Confocal Microscope. Z-stacks of the cell images were acquired; the optical slices in which the DAPI is most conspicuous (indicating slices at the intracellular level) were selected for analysis. The optical thickness for each slice was 2.51 µm and 0.47 µm for 20X and 60X magnification, respectively. Images were processed using the Nikon-Elements Advance Software or ImageJ (39, 40).

The methods for KRAS sequencing and rabbit polyclonal anti-KRAS antibody dialysis is in the **Supplementary Methods**.

## 3 Results

### 3.1 Establishment of transient *ex vivo* culture of CRC tumor and matched mucosa tissues

**Figure 1:**
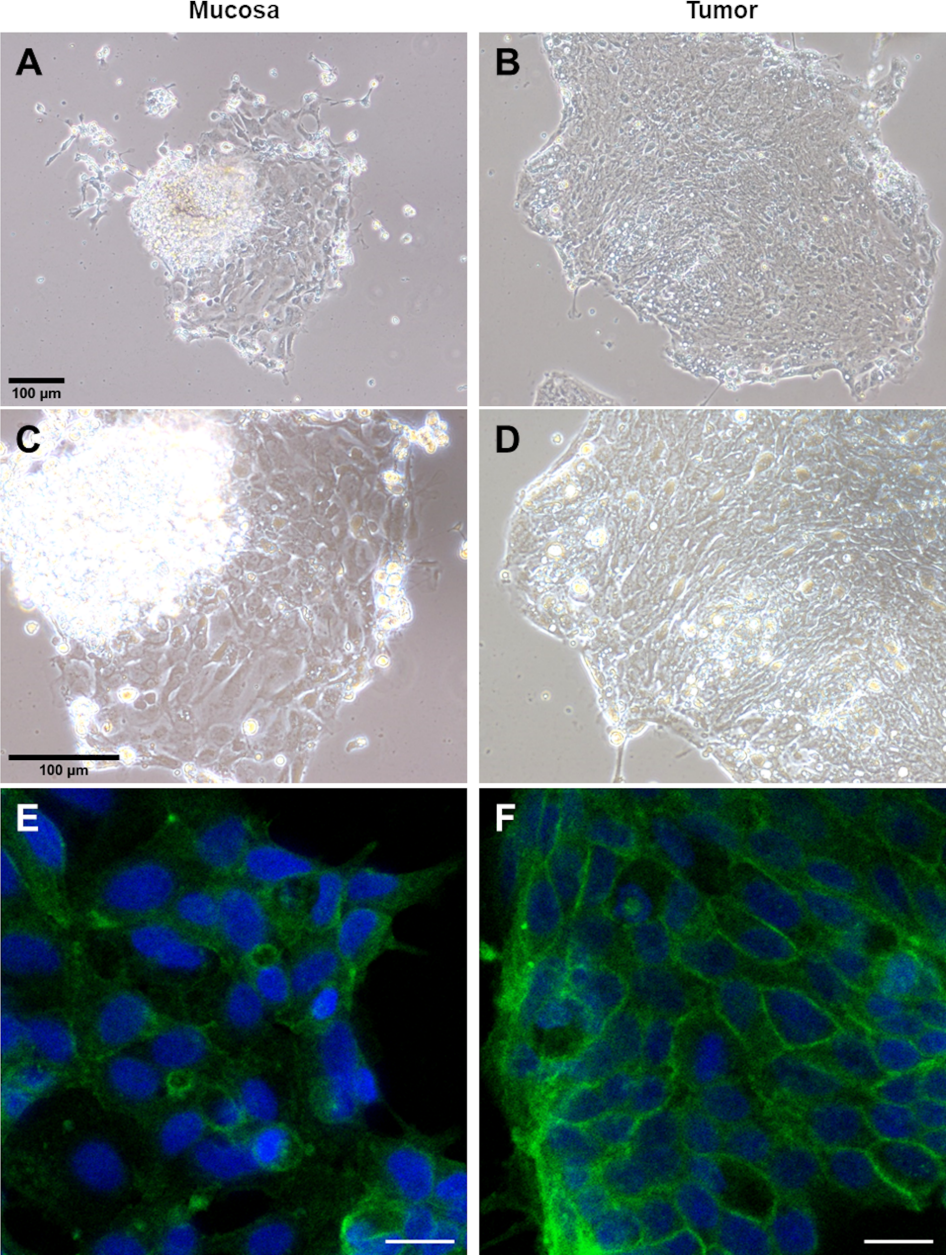
Crypt epithelial cells from patient-derived CRC matched mucosa and tumor tissues can be maintained and immunoassayed in *ex vivo* culture. Phase contrast images of a patient’s matched mucosa **(A, C)** and tumor **(B, D)** crypt epithelial cells at 10X **(A, B)** and 20X **(C, D)** magnification. Confocal imaging for *ex vivo* cultured colorectal mucosa **(E)** and tumor **(F)** cells which were fixed and immunostained for KRAS. KRAS is more evenly distributed in the cytoplasm in fixed *ex vivo* cultured mucosa cells **(E)** while localized more to the plasma membrane in tumor cells **(F)**. The tumor in **(F)** harbors a KRAS p.Gly12Val somatic mutation. The scale bar in **(A)** is applicable to **(A, B)** and in **(C)** is applicable to **(C, D)**. The scale bar in **(E, F)** is 20 µm.

To establish an *ex vivo* primary culture of both tumor and mucosa epithelial cells derived from freshly resected colorectal tissues of CRC patients, we first dislodged whole crypts from the tumor and matched mucosa tissues. The dislodged mucosa crypts appear to have a consistent elongated shaped ~200-300 µm (**Supplementary Figure 1C**) while the tumor crypts appear to be clumps of irregular shapes with varying sizes (**Supplementary Figure 1D**). This matches the common histological observation of mucosa having defined, organized and elongated crypts, while tumor having disorganized, often convoluted and irregularly shaped crypts (**Supplementary Figure 2**). Furthermore, the number of full complete crypts in tumor is visibly lower as compared to its’ matched mucosa (**Supplementary Figure 1**). To culture crypts epithelial cells, isolated crypts were seeded onto Matrigel-coated coverslips and incubated in cell culture medium supplemented with FBS without additional growth factors. The tumor and matched mucosa crypts epithelial cells were observed to be able to attach and spread on Matrigel-coated coverslips a day after seeding (**Figures 1A–D**). The mucosa crypt epithelial cells appear flatter, more widespread (**Figures 1A–C**) while the tumor epithelial crypt cells appear more tightly packed (**Figures 1B–D**). The *KRAS* mutation status of all *ex vivo* cultured specimens (n=70) are listed in **Supplementary Table 2**.

### 3.2 KRAS membrane localization observed in ex. vivo cultured and post-fixed KRAS p.Gly12Val tumors

We first determined the subcellular localization of KRAS in the *ex vivo* tumor and matched mucosa cells. Immunofluorescence (IF) staining revealed that KRAS localized predominantly to the cytoplasm in the matched mucosa (**Figure 1E**) and KRAS wild-type (WT) tumor (**Supplementary Figure 3**). However, KRAS were shown to localize largely at the inner plasma membrane resembling a net-like pattern in the KRAS p.Gly12Val (G12V) tumor (**Figure 1F**). Activated KRAS is expected to be tethered to the inner plasma membrane to activate downstream targets (e.g. BRAF), thus higher pools of inner membrane localized-KRAS indicates higher activation (41). This corroborates with the concept that KRAS oncogenic mutants (but not WT) are constitutively activated. KRAS inner plasma membrane localization in tumor with *KRAS* mutation is also consistent with the observation in *KRAS* mutated CRC cell lines like SW480 [Figure 6C of Zhang, Jiang (42)].

### 3.3 Anti-KRAS antibody can be internalized into live colon mucosa and tumor cells and form punctate structures

**Figure 2:**
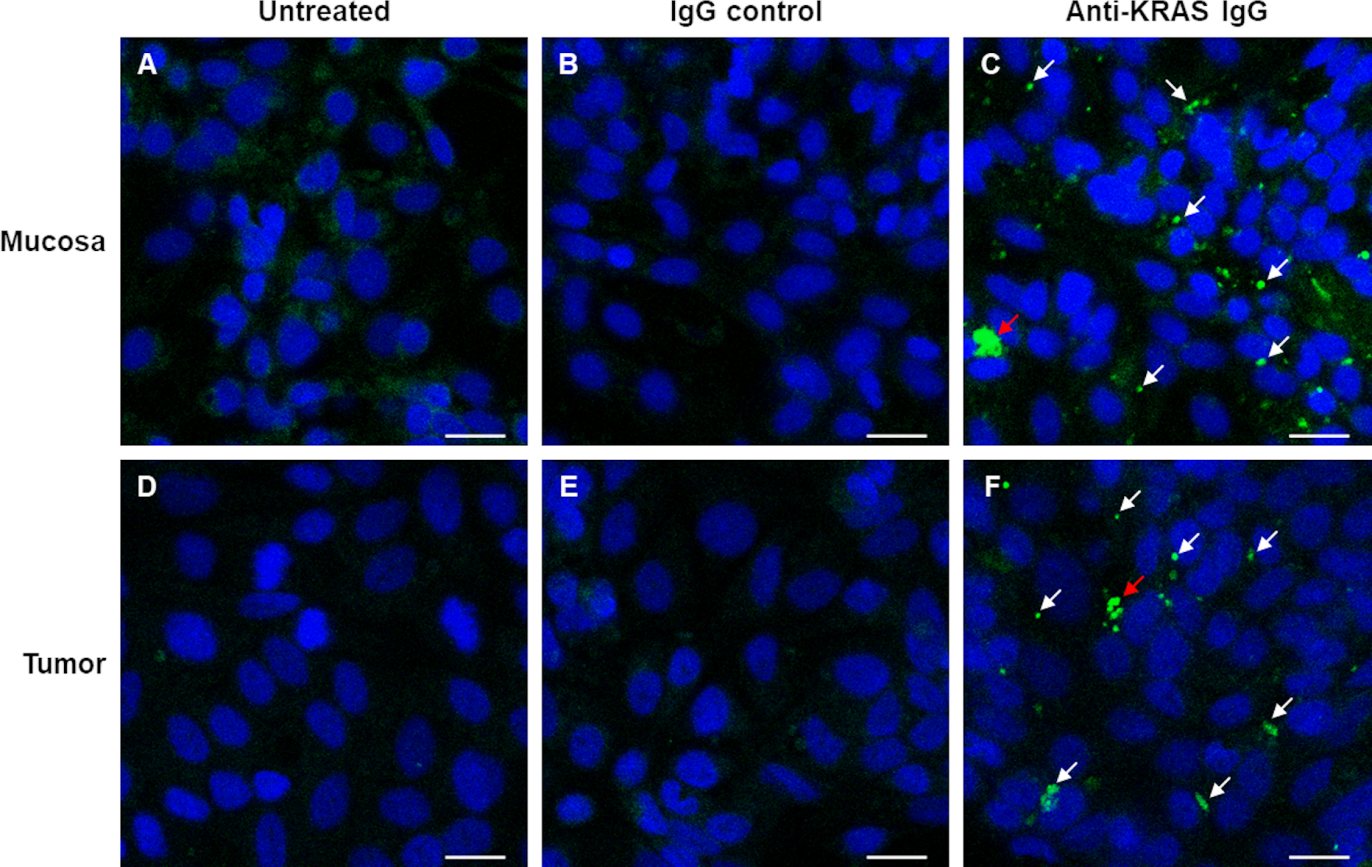
Confocal imaging shows internalization of anti-KRAS antibodies into *ex vivo* cultured mucosa and tumor cells. Live *ex vivo* cultured matched mucosa **(A–C)** and tumor **(D–F)** cells were untreated **(A–D)**, treated with Rabbit IgG Isotype control **(B–E)** or Rabbit anti-KRAS antibody **(C–F)** for 16 h, then fixed and counterstained with FITC-conjugated anti-rabbit IgG secondary antibody. The arrows in **(C)** and **(F)** point to the aggregates of anti-KRAS antibodies; the red arrows point to larger aggregates. Scale bar = 20 µm.

Since KRAS proteins are inside the cancer cells, anti-KRAS antibodies must be able to enter the cytoplasm to bind to the KRAS molecules. Thus, we assessed whether anti-KRAS antibody can be internalized into live *ex-vivo* cultured mucosa-tumor pairs. Live *ex vivo* cultured cells were treated with rabbit anti-KRAS IgG antibody, rabbit IgG isotype control, or antibody diluent for 16 h at 33°C, fixed and counterstained with FITC-conjugated secondary antibodies. As shown in **Figure 2** (Panel C and F), anti-KRAS antibody was internalized into the cytoplasm and formed punctate structures in both the mucosa and tumor. No punctate structures could be observed in the untreated (**Figures 2A–D**) and rabbit IgG control antibody-treated (**Figures 2B–E**) live mucosa-tumor cells. The punctate staining indicates that the anti-KRAS antibody was internalized into both the mucosal and tumor cells. These punctate staining were not observed in cells treated with Rabbit IgG isotype control, further supporting that the punctate structures were likely due to KRAS-specific antibody-protein complexes formation. Co-localization of two different anti-KRAS antibodies in the same structures provided additional evidence that the aggregates were KRAS-antibody complexes (**Supplementary Figure 4**).

### 3.4 The internalized anti-KRAS antibodies were not accumulated in the endosomes

It is possible that a significant pool of anti-KRAS antibody molecules internalized *via* the fluid-phase endocytosis pathway and thus may be trapped in endosomes. For the anti-KRAS antibodies to be able to bind to and sequester endogenous KRAS, they must be localized to the cytoplasm. However, endosomal escape is usually inefficient, leading to most cargoes end up being degraded in the lysosomes. It is possible that the punctate structures that we observed in **Figure 2** were due to accumulation of internalized anti-KRAS antibodies in the endosomes. To exclude this possibility, we treated live *ex vivo* cultured colon tumor cells with rabbit anti-KRAS antibodies. At 16 h after treatment, the cells were permeabilized and immunostained for early endosomal marker EEA1. Confocal imaging showed that most of the anti-KRAS antibody-positive punctate structures did not co-localize with EEA1 (**Supplementary Figure 5A-C**), excluding the possibility that the internalized antibodies were accumulated in the endosomes.

### 3.5 Tumor cells with KRAS p.Gly12Val mutation showed reduced KRAS membrane localization after anti-KRAS antibody treatment

**Figure 3:**
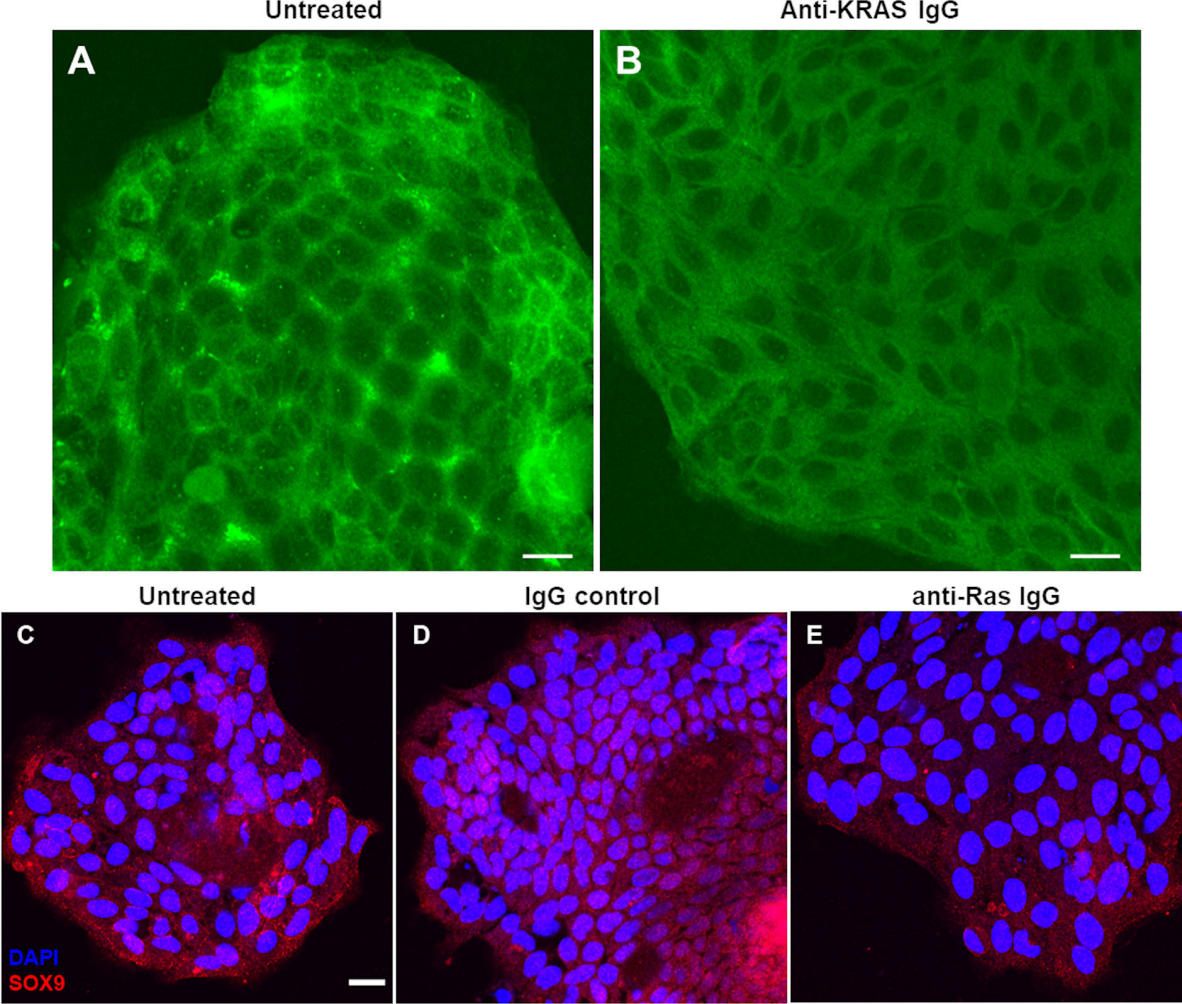
Decreased KRAS inner membrane localization and reduced SOX9 immunostaining after treatment of live *ex vivo* cultured tumor cells with anti-KRAS antibody observed by confocal imaging. Live *ex vivo* cultured tumor cells were untreated (negative control) **(A)** or treated with mouse anti-KRAS antibody **(B)**, fixed and immunostained with rabbit-anti-KRAS antibody then counterstained with FITC-conjugated anti-rabbit IgG secondary antibody. Live *ex vivo* cultured tumor cells were untreated **(C)** or treated with rabbit IgG isotype control **(D)** or rabbit anti-Ras IgG antibody **(E)**, fixed and immunostained with mouse anti-SOX9 antibody then counterstained with Alexa Fluor 555-conjugated anti-mouse IgG secondary antibody. The tumor in **(A, B)** harbors a KRAS p.Gly12Val mutation. The scale bar in **(A–C)** are equivalent to 20 µm. The scale bar in **(C)** is applicable to **(C–E)**.

We hypothesize that treatment of *ex vivo* cultured tumor with anti-KRAS antibody can reduce the membrane localization of KRAS by aggregating KRAS in the cytoplasm. We treated the KRAS p.Gly12Val tumor cells with mouse anti-KRAS antibody, then fixed and immunostained for KRAS using rabbit anti-KRAS antibodies. Compared to untreated tumor control (**Figure 3A**), treated tumor cells has reduced KRAS plasma membrane localization (**Figure 3B**). Meanwhile, KRAS wild-type tumor cells do not show obvious alterations in KRAS localization (data not shown).

### 3.6 *Ex vivo* cultured tumor treated with anti-KRAS antibodies displayed decreased SOX9 expression and nuclear localization

While treatment of tumor cells with anti-KRAS antibodies led to an observable change of localization of KRAS from inner plasma membrane to cytoplasm, its effect on downstream signaling remains to be confirmed. Phospho-ERK1/2 and phospho-Akt1/2/3 are known downstream effectors of KRAS in the MAPK signaling in cancer cell lines (21). However, we previously reported that phospho-ERK1/2 or phospho-Akt1/2/3 were not upregulated in CRC tumor compared to matched mucosa tissues, and in fact in most cases, downregulated in tumor (24). Instead, most CRC tumor tissues displayed upregulation of SOX9 compared to matched normal mucosa. Furthermore, SOX9 were previously reported to be able to promote cancer cell proliferation and tumor progression by directly activating stem cell-like signaling and inhibit cell differentiation (43). Since we showed previously that the expression of SOX9 proteins in CRC was KRAS-mutant-dependent (24), treatment of CRC with anti-KRAS antibodies is expected to downregulate SOX9 protein expression. Thus, we counterstained the tumor cells for SOX9 after treatment with anti-KRAS antibodies. Compared to untreated cells (**Figure 3C**) or tumor cells treated with rabbit IgG negative control (**Figure 3D**), tumor cells treated with anti-KRAS antibody showed weaker SOX9 cytoplasmic staining and less nuclear localization (**Figure 3E**).

## 4 Discussion

Direct inhibition of KRAS has been a longstanding challenge, due to its structure being impermissible to pharmacological targeting. While it is largely believed that macromolecules above 1 kDa cannot penetrate the cell membrane, the existence of cell-penetrating autoantibodies of ~150 kDa contradicted this concept (26, 44). We thus speculated that anti-KRAS antibodies can similarly penetrate cells and inhibit endogenous KRAS.

In this study, we developed a transient *ex vivo* culture from CRC patients’ tumor and matched mucosa tissues (**Figures 1A–D**) as a model to test for anti-KRAS antibody internalization. We first characterized the localization of endogenous KRAS in fixed and permeabilized *ex vivo* cultured CRC matched mucosa-tumor pair which revealed that tumors with somatic KRAS p.Gly12Val activating mutations show higher inner plasma membrane KRAS localization (net-like pattern) compared to its matched mucosa (where KRAS was wild-type) (**Figures 1E, F**). This is possibly contributed by increased KRAS activation hence increase membrane dwell time which is essential for KRAS effector function (41). Interestingly, the net-like pattern is less prominent in other KRAS activating mutation tumors, which may be due to different plasma membrane anchoring properties of the mutants.

We next hypothesized that anti-KRAS antibodies can penetrate cells and enter the cytosol to sequester endogenous KRAS, preventing its plasma membrane localization. Our results show that anti-KRAS antibody can indeed be internalized into both *ex vivo* cultured matched mucosa and tumor cells (**Figures 2C–F**). We observed that the internalized anti-KRAS antibodies form aggregates (punctate structures) in the cells (**Figures 2C–F**), but not in untreated (**Figures 2A–D**) or cells treated with rabbit IgG isotype control (**Figures 2B–E**), showing that the aggregates are not due to fortuitous antibody aggregation but possibly aggregation of endogenous KRAS by anti-KRAS antibodies. The aggregates were confirmed to be KRAS-antibody complexes with co-localization of two different anti-KRAS antibodies (**Supplementary Figure 4**).

These antibodies are largely localized in the cytoplasm with little trapped in endosomes as observed from minimal co-localization of the internalized anti-KRAS antibody with early endosome marker EEA1 (**Supplementary Figure 5**). The internalized anti-KRAS antibodies binds to and aggregates endogenous KRAS in the cytoplasm. Shin, Choi (45) and Shin, Kim (46) has claimed that entry of anti-Ras antibody into cell line was mainly by endocytosis with endosomal escape efficiency at only 4-5% and 13-16% respectively while most antibodies are trapped in endosomes. Our results may be attributed to different cells, different antibodies, or HEPES in our antibody dilution medium which has been reported to drive protein transduction, or components from the Matrigel that may mediate alternative non-endocytic entry (47).

Treatment of live *ex vivo* cultured tumor cells harboring p.Gly12Val somatic mutations with anti-KRAS antibodies reduced inner plasma membrane localization of KRAS where it must tether to function (**Figure 1F**). Live tumor cells treated with anti-KRAS antibody also showed less intense cytoplasmic and less nuclear staining for SOX9, thus providing further evidence that SOX9, a cancer stem cell marker and potential driver in CRC (43, 48), is a downstream effector of KRAS signaling (**Figures 3C–E**). These differential phenotypes of anti-KRAS antibody-treated tumor cells indicated that the internalized antibody is functional and could potentially be developed into novel antibody therapeutics for CRC.

**Figure 4:**
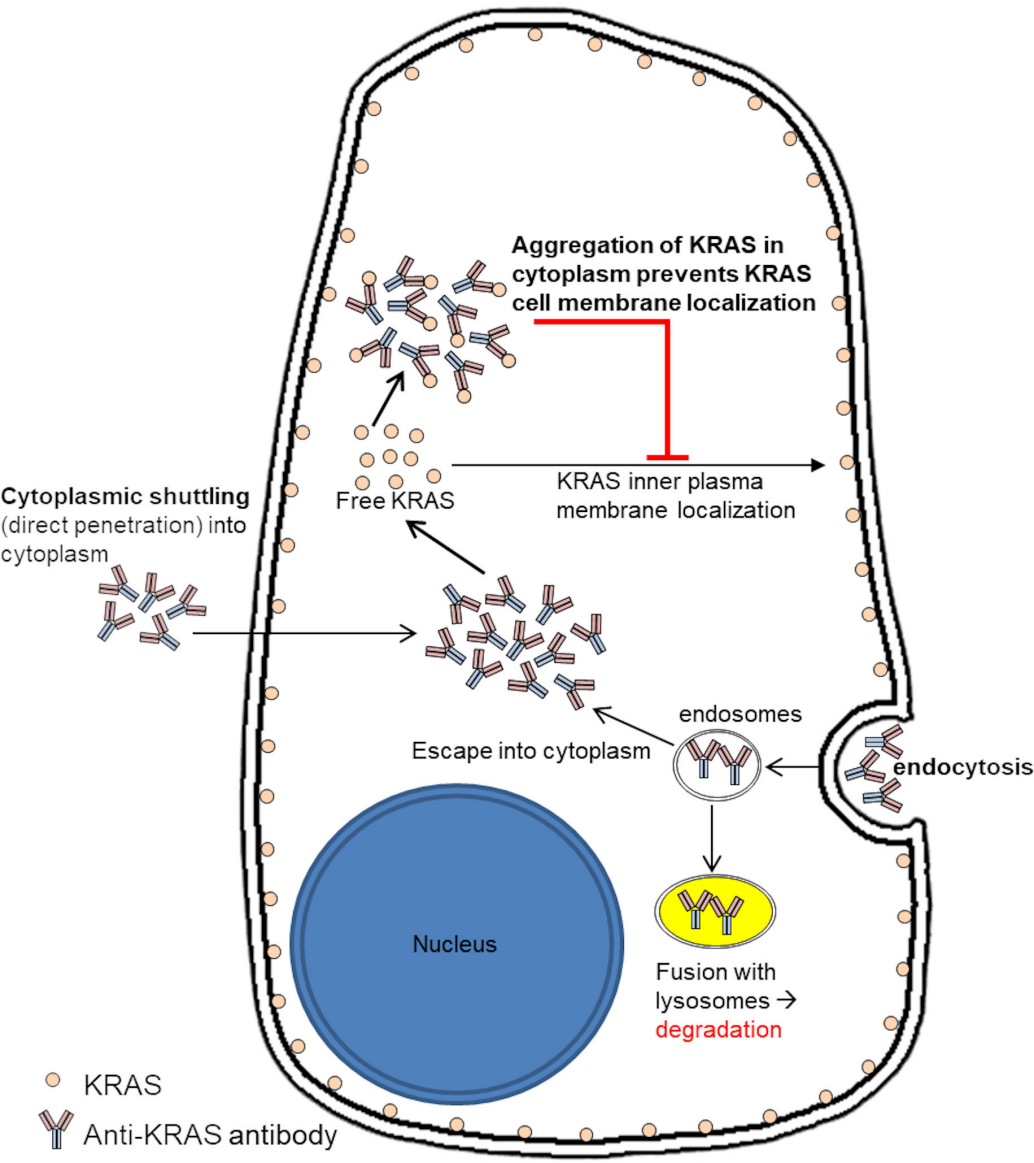
Proposed mode of action of anti-KRAS antibody treatment on sequestering endogenous KRAS in the cytoplasm to inhibit them from localizing to the inner plasma membrane. We propose that anti-KRAS antibodies taken up by the cells enters the cytoplasm and binds to endogenous KRAS, preventing KRAS from localizing to the inner plasma membrane where it activates downstream effectors.

We propose a mode of action of anti-KRAS antibody in inhibition of KRAS signaling and inner plasma membrane localization (**Figure 4**). Anti-KRAS antibodies must first be internalized and localized to the cytoplasm, either by fluid-phase endocytosis then endosomal escape or by direct penetration through the plasma membrane lipid bilayer. The antibodies then bind to endogenous KRAS in the cytoplasm and prevent KRAS from being translocated to the inner plasma membrane where it functions.

While small molecules are the major trend in therapeutics due to their small size, thus easy entry into cells, macromolecule biologics like antibodies have several advantages. Small molecules tend to have off-target pleiotropic effects, whereas antibodies, a type of biologics, are more specific for their target due to a higher structure complexity (49). While monobodies can also target internal proteins, they lack the Fc domain of intracellular antibodies which can be recognized by cytosolic Fc receptor to activate the intracellular immunity through TRIM21 and targets the antibody-protein complex for degradation (50–52). Since IgG antibodies are naturally occurring, toxicity usually associated with small molecule inhibitors and monobodies is minimized. Moreover, if the mode of action is mislocalization rather than altered expression of the protein, therapy-induced secondary mutations and resistance can conceivably be reduced.

The long-term goal is to produce antibodies that specifically target mutant KRAS. The antibodies should be further optimized for more efficient internalization by tumor cells, with the aim of eventually proceeding to clinical trials. Anti-KRAS antibodies can be used in combination with anti-EGFR antibody (Cetuximab) in *KRAS* wildtype advanced colorectal cancer (CRC) patients to concurrently kill tumor cells that has developed resistance to anti-EGFR therapy due to acquired KRAS mutation. For patients who are already *KRAS* mutation-positive, anti-KRAS antibody targeting the hot-spots codons-12 and -13, will be the therapy of choice. Furthermore, majority of pancreatic ductal adenocarcinoma (PDAC), another common fatal cancer, is initiated by *KRAS* mutation and hence overexpression of the KRAS protein (53). Thus, the successful production of the anti-KRAS antibody targeting the mutational hot spots will potentially be a useful alternative therapy for PDAC as well.

## Data availability statement

The raw data supporting the conclusions of this article will be made available by the authors, without undue reservation.

## Ethics statement

The studies involving human participants were reviewed and approved by Singhealth Centralized Review Board. The patients/participants provided their written informed consent to participate in this study.

## Author contributions

PC and SW contributed to the concept and study design. KL and YL contributed to the experimental planning and the acquisition, analysis, and interpretation of data. CT, ET, AC and IS-E contributed to the tissue collection, clinical data and administration. ML and MW contributed to the tissue collection process and ML contributed to execution of certain experimental procedures. KL, YL and PC drafted the manuscript. KL, PC and SW contributed to the critical revision of the manuscript. All authors contributed to the article and approved the submitted version.
