# Supplementary material for: KRAS-specific antibody binds to KRAS protein inside colorectal adenocarcinoma cells and inhibits its localization to the plasma membrane
Source: Front Oncol. 2023 Mar 27;13:1036871. doi: 10.3389/fonc.2023.1036871 (PMC10084885; doi:10.3389/fonc.2023.1036871)
Supplement: Supplementary file 1 [file DataSheet_1.docx]

Supplementary Material

**KRAS-specific antibody binds to KRAS protein inside colorectal adenocarcinoma cells and inhibits its localization to the plasma membrane**

**Supplementary Figure 1.** Tumor and matched mucosa tissues from freshly resected colorectal specimens, the dotted lines indicate the tissue excised for *ex vivo* culture (A). Three portions of colorectal mucosa crypts dislodged with different chelating buffer (B). Brightfield images of bottom region of crypts dislodged from patient-derived CRC mucosa (C) and tumor (D) tissues. The scale bar in (C) is applicable to (C, D)

**Supplementary Figure 2.** **Toluidine blue staining of cryosections of a pair of colorectal matched mucosa and tumor.** The crypts in the mucosa (A) are elongated, organized and regularly sized, while the crypts in the tumor (B) are distorted and vary in sizes.

**Supplementary Figure 3. Confocal imaging for ex vivo cultured matched colorectal mucosa (A) and KRAS WT tumor cells (B) which were fixed and immunostained for KRAS.** Scale bar =20 µm.

**Supplementary Figure 4:** Co-localization of internalized mouse monoclonal anti-KRAS immunostaining (red) in live tumor cells followed by rabbit polyclonal anti-KRAS immunostaining (green) of the same tumor cells post-fixation, viewed in the TRITC (A), FITC (B), Merged channels (C and D). Nuclei were stained with DAPI (blue). The antibodies overlap (yellow) in some but not all punctate structures. Steric hindrance could have prevented co-localization in other structures.

**Supplementary Figure 5. Confocal imaging shows some internalized anti-KRAS antibodies were in the early endosomes but most were distributed in the cytoplasm**. Live *ex vivo* culture tumor cells were treated with rabbit anti-KRAS for 16 h, fixed and immunostained with mouse anti-EEA1 IgG, then counterstained with Alexa fluor 555-conjugated anti-mouse IgG (A); the same section was simultaneously counterstained with FITC-conjugated anti-rabbit IgG to visualize the internalized anti-KRAS antibody (B). The merged image shows minimal overlap of EEA1 and anti-KRAS antibody. The arrow indicates overlap between EEA1 and internalized anti-KRAS antibody (C). Scale bar = 10 µm.

**Supplementary Table 1.** Primary antibodies used for treatment or post-fixation immunostaining of *ex vivo* culture cells

| **Antibody** | **Brand / Catalogue number** | **Immunogen** |
| --- | --- | --- |
| Rabbit Polyclonal anti-KRAS antibody IgG^a^ | Thermo Fisher Scientific / PA5-27234 | KRAS 1-72 aa |
| Mouse monoclonal anti-KRAS Antibody (9.13) IgG1 | Thermo Fisher Scientific / 415700 | KRAS 4A 168-180 aa |
| Rabbit monoclonal anti-Ras IgG^b^ | Cell Signaling Technology / E4K9L | KRAS residues surrounding Glu37 |
| Mouse monoclonal anti-EEA1 antibody [1G11] IgG1 | Abcam / ab70521 | EEA1 50 aa to end of C-terminus |
| Mouse monoclonal anti-SOX9 antibody IgG2a | Sigma-Aldrich / AMAB90795 | SOX9 42-158 aa |

aa, amino acid

^a^ Dialyzed in PBS to remove ProClin™ 300 preservative

^b^ Provided in carrier-free lyophilized format, reconstituted in PBS without preservatives

**Supplementary Table 2.** KRAS mutation status of tumors successfully cultured

| **KRAS mutations** | **Count** | **%** |
| --- | --- | --- |
| Wild-type | 43 | 61.43% |
| p.Gly12Val | 11 | 15.71% |
| p.Gly12Asp | 6 | 8.57% |
| p.Gly12Ala | 1 | 1.43% |
| p.Gly12Cys | 1 | 1.43% |
| p.Gly12Tyr | 1 | 1.43% |
| p.Gly13Asp | 5 | 7.14% |
| p.Gly49X | 1 | 1.43% |
| p.Lys117Asn | 1 | 1.43% |
| **Total** | **70** | **100%** |

**Supplementary Table 3.** Primers used for PCR amplification and sequencing of KRAS

| **Target** | **Primer** | **Sequence** |
| --- | --- | --- |
| KRAS Exon 2 | KRAS Ex2(-185)F  KRAS Ex2(+198)R | TCATTACGATACACGTCTGCAG  CCCTGACATACTCCCAAGG |
| KRAS Exon 3 | KRAS Ex3(-169)F  KRAS Ex3(+173)R | TTCAGGTGCTTAGTGGCC  CACTGCTCTAATCCCCCAAG |
| KRAS Exon 4 | KRAS Ex4(-208)F  KRAS Ex4(+175)R | TTGATCTTTTGAGAGAGATACAAGG  GCAGTACCATGGACACTGG |

**Supplementary Methods**

## Genomic DNA extraction and KRAS Exons sequencing of tumor samples used for ex vivo culture

Tumor tissues were enriched for neoplastic cells (at least 90%) by macro-dissection and genomic DNA was extracted with the DNeasy Blood & Tissue Mini Kit (Qiagen, Hilden, Germany). *KRAS* Exons 2, 3, 4 harboring the coding sequence were PCR amplified from patient tumor genomic DNA using GoTaq® Flexi DNA Polymerase Kit (Promega, Wisconsin, U.S.A). The accession number of the RefSeq Human KRAS sequence is NM_033360.4 and the NCBI Gene ID is 3845. The PCR products were purified by isopropanol precipitation or column purification. Sequencing was performed with Applied Biosystems (Massachusetts, U.S.A) BigDye™ Terminator v3.1 Cycle Sequencing Kit using the PCR products as templates. The reaction was purified by ethanol precipitation and resuspended in Hi-Di™ Formamide, then separated by capillary electrophoresis in 3500 Genetic Analyzer (Applied Biosystems). The output sequencing results were visualized on Chromas v2.6.4 (Technelysium Pty Ltd, South Brisbane, Australia) as chromatograms and visually inspected for mutations, the sequences were also BLAST (1) against the RefSeq human *KRAS* sequence. Both PCR and Sanger sequencing reactions were performed on Biometra (Analytik Jena AG, Jena, Germany) UNOII or GeneAmp 9700 (Applied Biosystems) Thermocyclers. All primers are listed in Supplementary Table 3.

## Rabbit anti-KRAS antibody dialysis

The rabbit polyclonal anti-KRAS antibody (Thermo Fisher Scientific cat# PA5-27234) was dialyzed using the gamma-irradiated, 10K MWCO Slide-A Lyzer™ G2 Dialysis Cassette (Thermo Fisher Scientific) according to manufacturer’s protocol to remove ProClin™ 300 from the antibody stock. The cassette was immersed in the dialysis buffer PBS for 2 minutes. 280 µL of antibody was loaded into the cassette using a 23-gauge, 1 mL syringe penetrated into the gasket cassette. The antibody underwent 4 changes of dialysis buffer, 1 L of fresh PBS for 2 h at 4 °C each round for the first 3 rounds, then 1 L of fresh PBS for 16 h at 4°C. The dialyzed antibody was recovered and the protein concentration was determined with Nanodrop A280 module (Applied Biosystems, MA, USA).

**References**

1. Altschul SF, Gish W, Miller W, Myers EW, Lipman DJ. Basic Local Alignment Search Tool. *J Mol Biol* (1990) 215(3):403-10. Epub 1990/10/05. doi: 10.1016/S0022-2836(05)80360-2.
